# E161111 is an ultra-short-acting etomidate analogue with stable haemodynamics that elicits only slight adrenocortical suppression in rats

**DOI:** 10.7717/peerj.13492

**Published:** 2022-05-24

**Authors:** Bin Wang, Deying Gong, Yi Kang, Jin Liu, Jun Yang, Wen-sheng Zhang

**Affiliations:** 1 Department of Anesthesiology, People’s Hospital of Guizhou Province, Guiyang, Guizhou, China; 2 Laboratory of Anaesthesia and Critical Care Medicine, Translational Neuroscience Center, West China Hospital, Sichuan University, Chengdu, Sichuan, China; 3 National-Local Joint Engineering Research Center of Translational Medicine of Anesthesiology, West China Hospital, Sichuan University, Chengdu, Sichuan, China

**Keywords:** Etomidate analogue, Soft drug, Adrenocortical suppression, Ultra-short-acting, Continuous infusion, Stable haemodynamics

## Abstract

**Purpose:**

We report on a novel ultra-short-acting etomidate analogue, E161111, which has the same primary metabolite as etomidate.

**Methods:**

The metabolic rate of E161111 was determined in rat plasma and liver homogenate. Rats were infused for 30 or 60 min to maintain light sedation at Richmond Agitation-Sedation Scale (RASS) for −2 to 0 score. Mean arterial pressure (MAP) was monitored during 30 min infusion. The serum corticosterone was determined during and 3 h after infusion as a measure of adrenocortical function.

**Results:**

E161111 was not detected in rat plasma at 1 min (t_1/2_ = 6.69 ± 0.07 s) and in rat liver homogenates at 5 min (t_1/2_ = 10.20 ± 3.76 s); its main metabolic product was etomidate acid. The recovery time from loss of righting reflex (LORR) was 4.3 ± 1.5 min after 1-h infusion of E161111. During 30 min infusion, E161111 did not cause MAP changes. The stimulated serum corticosterone levels after 1-h infusion of E161111 were significantly higher than that after 1-h infusion of etomidate at all time points tested for the 3 h study.

**Conclusions:**

E161111 was metabolised rapidly, the metabolites were same as etomidate, and the recovery time after 1-h infusion was short. It elicited haemodynamic stability and milder suppression of corticosterone than that elicited by etomidate.

## Introduction

Etomidate is unsuitable for administration by a prolonged infusion due to its adrenocortical suppression and long recovery time. Some scholars have reported that single bolus etomidate administration or long-term infusion with etomidate can increase the risk of mortality due to its inhibitory effect on adrenocortical function, especially for patients undergoing intensive care (Chan, Mitchell & Shorr, 2012; Sunshine et al., 2013; Edalatkhah et al., 2021; Albert, Ariyan & Rather, 2011; Komatsu et al., 2013). Although the increased mortality associated with etomidate use is still controversial, the long time needed to recover from anaesthesia/sedation and muscle tremors after its infusion restrict its application. It takes 4 min to recover after a 5-min infusion, and 31 min after a 2-h infusion (Malapero et al., 2017). Therefore, it would be desirable to modify the chemical structure of etomidate to maintain its hemodynamic properties while limiting its adrenal cortical suppression and long-term recovery time.

Etomidate inhibits the 11β-hydroxylase and cholesterol side chain cleavage enzymes in adrenal steroidogenesis. Previous studies on etomidate analogues have demonstrated that modifying the imidazole ring or ester side chain may decrease the inhibitory effect of etomidate on adrenal cortical function, while not influencing hemodynamic stability (Ge et al., 2013; Ge et al., 2014; Santer et al., 2015; Wang et al., 2017; Jiang et al., 2017; Xu et al., 2021). In our previous study, ET-26 hydrochloride was proven to have mild adrenocortical suppression while keeping hemodynamic properties. However, due to its long recovery time, it is not suitable for prolonged infusion (Jiang et al., 2017).

In order to achieve the purpose of rapid decomposition, “soft drugs” (*i.e*., drugs that undergo predictable metabolism to inactive metabolites after exerting their therapeutic effect) have been designed. The common design feature is that an ester group could be rapidly hydrolyzed by esterases (Husain et al., 2012). The main metabolite of etomidate, etomidate acid, has a lower inhibitory effect on the steroid 11β-hydroxylase than etomidate and should be safer. We hypothesized that if a soft analogue of etomidate, synthesized with ester chain modification and metabolized to etomidate acid it would be metabolised quickly and largely reduce adrenocortical inhibition while retaining stable hemodynamic profile.

E161111 was designed based on above concepts, with a carbonate ester side chain that could be rapidly hydrolyzed. In order to examine our hypothesis, we examined the *in vitro* metabolic rate of E161111 in the plasma and liver tissue of rats. We observed the recovery time using the Richmond Agitation-Sedation Scale (RASS) −2 to 0 score after 1-h infusion. We also measured the level of cortical hormones during 1-h infusion and blood pressure during 0.5-h infusion of E161111 in rats compared with those of etomidate and propofol.

## Material and Methods

### Ethical approval of the study protocol

All animal studies were conducted within the rules and regulations set by Committee of Scientific Research and Institutional Animal Experimental Ethics Committee, West China Hospital within Sichuan University (Chengdu, China). The ethic number was 2015015A.

### Animals

Adult male rats (220–250 g) were purchased from Chengdu Dassy Biological Technology (Chengdu, China). They were housed in cages in a room in the Animal Experimental Center of Sichuan University. The ambient temperature of this room was 25 ± 1 °C, with controlled humidity of 60% and a 12-h light–dark cycle (7:00 am to 7:00 pm). Rats had access to water and food *ad libitum* and were allowed to acclimatise to their environment for 7 days before experimentation. Ten rats were implanted with transmitters (RAT-HD-S21; Data Sciences International, St Paul, MN, USA) into the abdominal aorta under 2–3% sevoflurane anesthesia, while a homoeothermic blanket was used to maintain the body temperature of the rats at 36–38 °C. The rest of the experimental interventions and sample collections were accomplished through the tail vein puncture. Thus, no anesthetic procedure was used. At the end of study, all rats were euthanized by overdose of pentobarbital sodium.

### Synthesis of E161111

A total of 1.29 g of chloromethyl chloroformate (CAS: 22128-62-7) and 0.66 g of isopropanol were added to 30 mL of anhydrous dichloromethane, and the reaction solution was cooled with cold water. Then, 1.6 g of pyridine was added dropwise to the solution, and the latter was stirred for 2 h. Next, 50 mL of dichloromethane was added. The organic layer was washed twice with 2 N hydrochloric acid, and then washed once with water. The organic layer was separated and dried with anhydrous sodium sulphate overnight. After filtration, the filtrate was evaporated to obtain 1.28 g of crude isopropyl carbonate chloromethyl ester, which was used directly for the next reaction.

**Figure 1:**
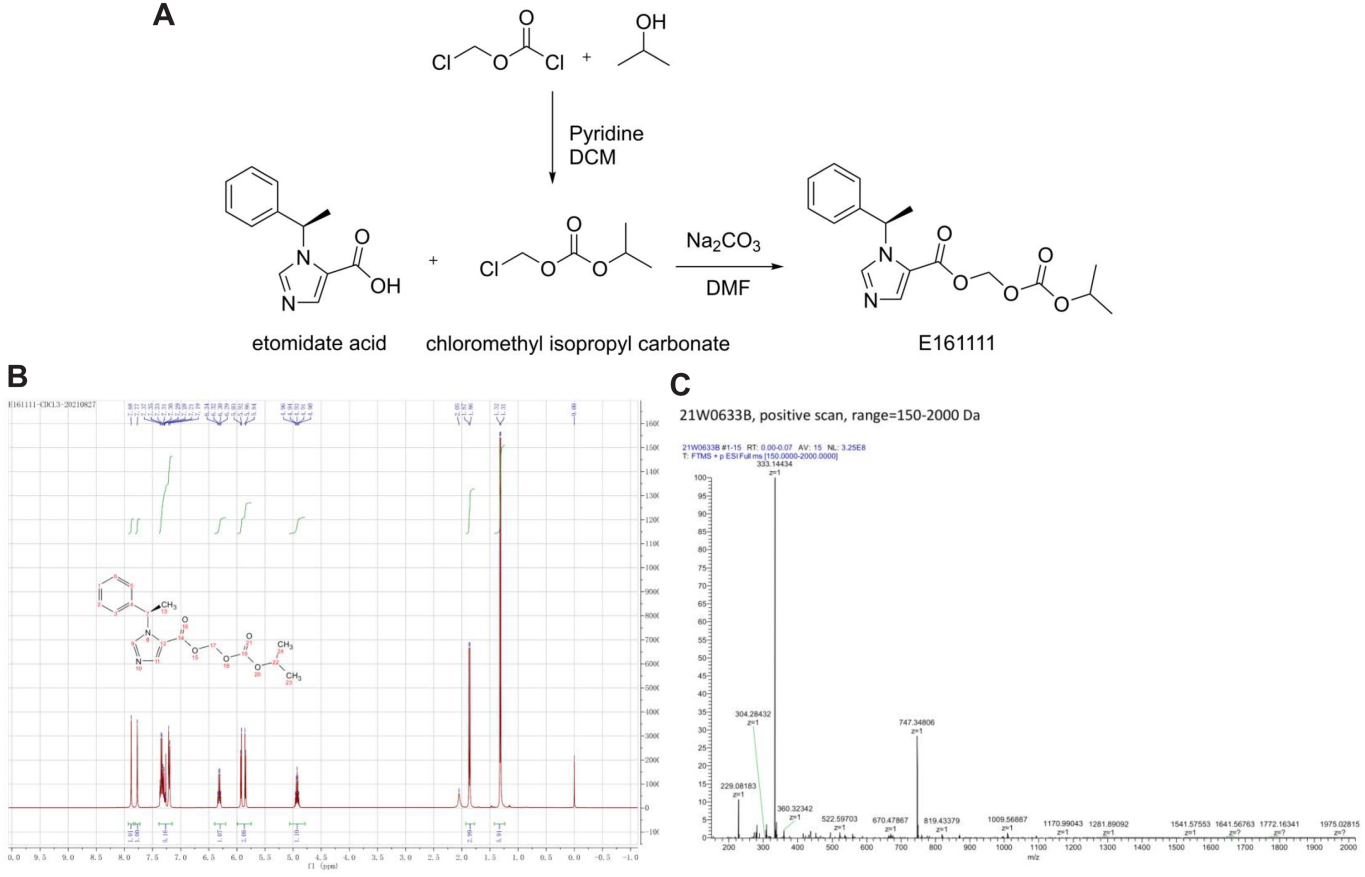
The structure and synthetic route of E161111 (A). 1H NMR and Mass spectrum of the E161111 (B, C). DCM, Dichloromethane; DMF, Dimethylformamide.

^1^HNMR (CDCl3, 400 MHz) δ:1.31 (6H, d, J = 4 Hz), 1.86 (3H, d, J = 8 Hz), 4.93 (1H, quint, J = 8 Hz), 5.85 (Ha, J = 8 Hz), 5.92 (Hb, J = 8 Hz), 6.30 (1H, q, J = 8 Hz), 7.19∼7.37 (5H, m), 7.77 (1H, s), 7.88 (1H, s); (Fig. 1B).

A total of 216 mg of etomidate acid was dissolved in 20 mL of dimethylformamide. Then, 155 mg of isopropyl carbonate chloromethyl ester and 310 mg of sodium carbonate were added to the solution. Next, the reaction solution was stirred for 3 h at room temperature. The reaction solution was filtered, 150 mL of water was added to the filtrate, and the mixture was extracted with 100 mL of dichloromethane. The organic layer was separated and dried overnight with anhydrous sodium sulfate. The solution was filtered and concentrated under reduced pressure to obtain a yellow oil. Finally, 266 mg of a white powder was obtained by column chromatography, and the yield was 80.1% (Fig. 1A).

HRMS calcd for C_17_H_20_N_2_O_5_ (M+H)^+^, 333.1372; found, 333.1443 (Fig. 1C).

### Metabolic rate in rat plasma and liver homogenate

Fresh rat plasma and liver tissues were obtained from three rats for experimentation using E161111. The mixed plasma or liver homogenate were added to samples to obtain an initial concentration of 1 μg/mL at 37 °C. At 0, 10, 20, 30 s, as well as 1, 2, 5, 10, 20, 40, and 60 min, 50-μL aliquots were pipetted, and the metabolic reaction was stopped with 150 μL of acetonitrile. After centrifugation at 20,000 rpm for 10 min at 4 °C, samples were analysed by high-performance liquid chromatography–mass spectrometry using a Mass-Hunter (1,260 series; Agilent Technologies, Santa Clara, CA, USA) system. The mobile phase consisted of methanol and water with 0.05% methanoic acid. The flow rate was 0.3 mL/min. An XSelect C18 (Waters, Milford, CT, USA) reversed-phase column (3.0 × 100 mm, 3.5 μm) was used. The spectrometer operated under the following conditions: polarity positive; capillary temperature = 350 °C; spray voltage = 45 psi; capillary voltage = 3,500 V; auxiliary gas flow = 5 L/min; 333.2 to 95 m/z; crash voltage = 86 V; collision energy = 8 V. The limit of quantification and limit of detection for E161111 was 0.05 μg/mL. The percentage of non-metabolised E161111 was calculated as the remaining compounds (μg/mL)/50 μg/mL. The metabolic half-life of E161111 was calculated using the equation: t_1/2_ = 0.693/Ke, Ct = C_0_ ·e^−ke.t^.

### Determination of the median effective dose (ED _50_), hypnotic and recovery assessment after hypnotic drug bolus

The Dixon and Mood up and down method (Dixon & Mood, 1948) was used to determine the ED_50_ for loss of righting reflex (LORR) after intravenous injection of E161111 diluted with normal saline to a solid volume of 0.3 mL as a bolus. If one rat showed LORR, a lower dose was given. A cross was used to indicate that LORR turned to no-LORR, or no-LORR to LORR. Once five of these crosses had been recorded, the test was terminated. The dose studied for E161111 ranged from 2.0 to 2.88 mg/kg with the actual doses given in 2.0, 2.4, 2.88 mg/kg. The ED_50_ was calculated as: ED_50_ = lg^−1^ (X_0_ + i (A/N ± 0.5)). The ED_50_ for LORR after one bolus of propofol or etomidate was obtained from previous study (Wang et al., 2017).

After administration of etomidate, E161111 and propofol (*N* = 10 rats for each group) at two folds of ED_50_ for LORR, time for duration of LORR and recovery from righting reflex to stand up were recorded.

### Infusion protocol and recovery observation

After determination of the ED_50_ for LORR, bolus administration of 1.2*ED_50_ was followed by a maintenance study after a 0.5 or 1-h continuous infusion. During these infusions, the infusion rate (in mg/kg·min) was adjusted to between 0.21 to 0.26 for etomidate, 0.77 to 0.94 for propofol and 4.29 to 5.04 for E161111 to maintain light sedation condition using a modified RASS score of −2 to 0 (Table 1) (Reade & Finfer, 2014). We define the light sedation as rats could be awaken with eye opening to voice.

**Table 1:** Sedation assessment and modified richmond agitation-sedation scale (RASS).

0 alert and calm	Alert and calm
-1 drowsy	Sustained awakening, ataxia and could not balance on hind limbs
-2 light sedation	Awakens with eye opening to voice, loss of righting reflex but with eyelash reflex
-3 moderation sedation	Loss of righting reflex with no eyelash reflex
-4 deep sedation	Response to physical stimulation
-5 cannot be aroused	No response to physical stimulation

Time for recovery from sedation condition to stand up were recorded after 1-h infusion of E161111, propofol or etomidate (*N* = 6 rats for each group). Adrenocortical studies were comparing after 1-h infusion of E161111 or etomidate (*N* = 9 rats for each group). Haemodynamic stability studies were comparing after 0.5-h infusion of E161111, propofol, or etomidate.

### Haemodynamic monitoring during 0.5-h infusion of a hypnotic agent

One week before infusion, rats underwent surgery as described previously (Wang et al., 2017). Ten rats were anaesthetised and then implanted with transmitters (RAT-HD-S21) of telemetry equipment (Data Sciences International, Saint Paul, MN, USA) into the abdominal aorta. These ten rats were used again after 1-week washout to make sure there were at least 6 rats in each group (two remaining rats in the second week were used for additional observation on new compound E161111): propofol (*N* = 6 rats), etomidate (*N* = 6 rats), and E161111 (*N* = 8 rats). Blood pressure was collected at baseline, as well as 1, 5, 10, 15, 25 and 30 min during 0.5-h infusion and 5, 10 min after infusion.

### Adrenocortical concentration after 1-h infusion of a hypnotic agent

Rats were divided into three groups of nine: normal saline, etomidate and E161111 (*N* = 9 rats for each group). A conventional adrenocorticotropic hormone (ACTH)-stimulation test at 25 µg/kg was used to measure the corticosterone concentration in serum. Dexamethasone (0.2 mg/kg) was administered 2 h before the test dose of study drugs were given at one bolus of 1.2*ED_50_ for LORR followed by a 1-h infusion. ACTH_1–24_ was administered 0.5 h before infusion, as well as 0.5, 1, and 2 h after infusion, to stimulate corticosterone production. Blood samples were collected at 2 h before, as well as 0, 0.5, 1, 2, and 3 h after infusion of test drugs. Serum samples were analyzed within 48 h using corticosterone ELISA kits (Biovendor, Brno, Czech Republic), as described previously (Wang et al., 2017).

### Statistical analyses

Data are the mean ± SD or ED_50_. The sample size was based on the precision observed from our previous studies. Plasma concentrations of corticosterone and mean arterial pressure (MAP) change were compared using two-way repeated-measures ANOVA followed by Tukey’s multiple comparison test. Analyses were carried out using SPSS 21 (IBM, Armonk, NY, USA). Graphs were created using Prism v7.0 (GraphPad, La Jolla, CA, USA).

## Results

### *In vitro* metabolism of E161111

**Figure 2:**
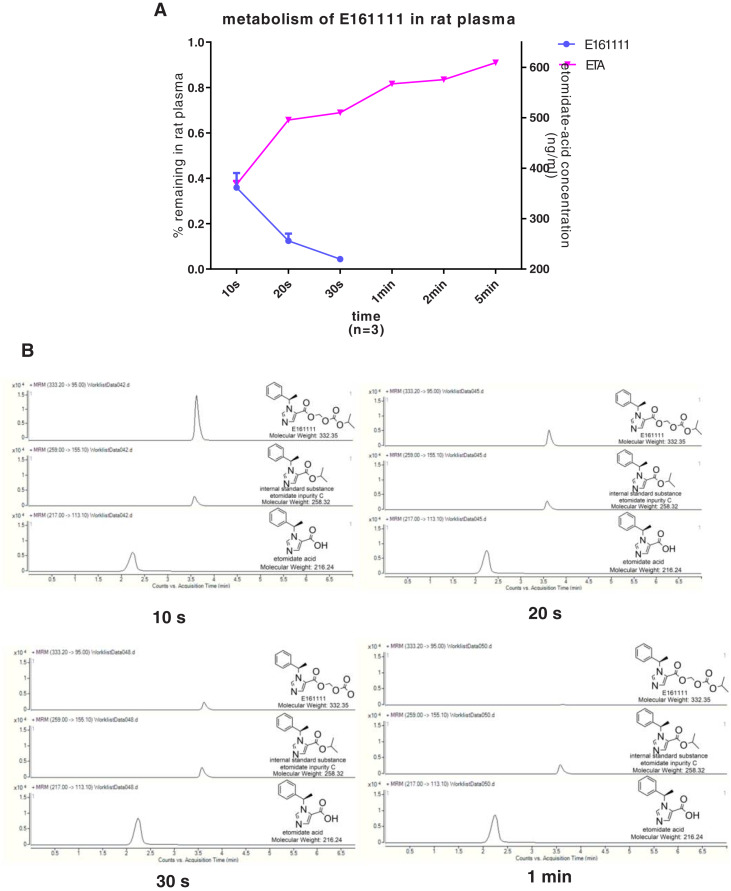
The percentage of remaining composition of E161111 (left x-axis) and etomidate-acid concentration in rat plasma (right y-axis, *n* = 3 rats) (A); mass spectra of E161111 and etomidate acid at 10, 20, 30 s, 1 min (B).

**Figure 3:**
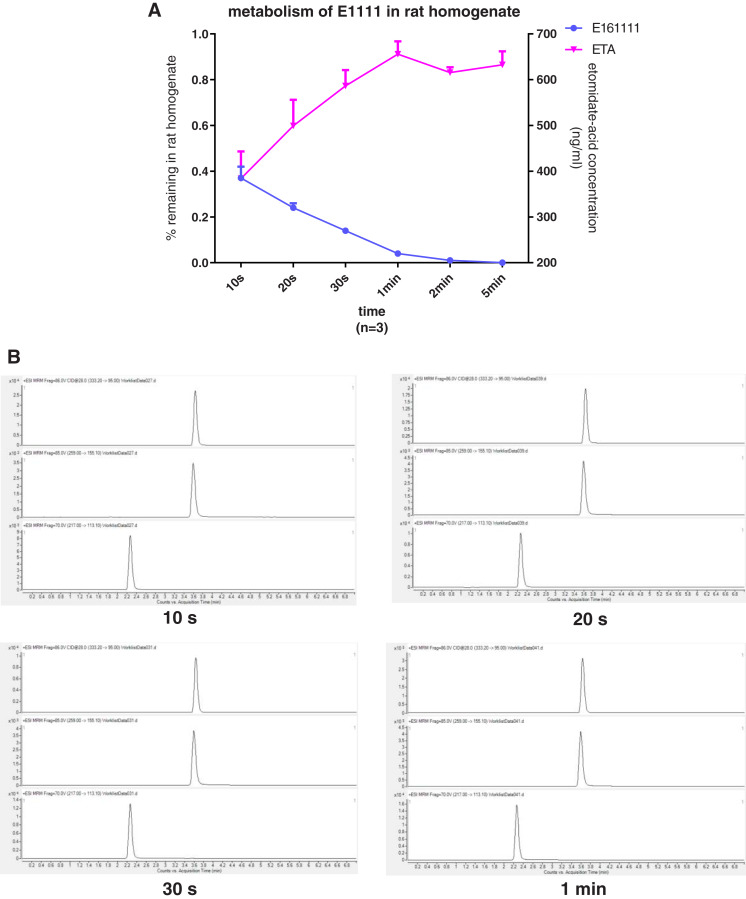
The percentage of remaining composition of E161111 (left x-axis) and etomidate-acid concentration in rat liver homogenate (right y-axis, *n* = 3 rats) (A); mass spectra of E161111 and etomidate acid at 10, 20, 30 s, 1 min (B).

We discovered that 4.39 ± 0.04% of undecomposed compounds could be detected after 30-s incubation in rat plasma and 14 ± 0.01% in rat liver homogenate. The primary metabolite, etomidate acid, had a concentration of 609.22 ± 3.43 ng/mL after 5-min incubation in rat plasma and 632.40 ± 29.60 ng/mL in rat liver homogenate (Figs. 2, 3). Theoretically, 65.1% of this concentration of E161111 would result in a concentration of etomidate acid of 651 ng/mL. The measured concentration of etomidate acid was in accordance with the theoretical value. The metabolic half-life for E161111 was 6.69 ± 0.07 s in rat plasma and 10.20 ± 3.76 s in rat liver homogenate.

### ED_50_ for LORR after one bolus administration and infusion rate between 1-h infusion

The potency (in mg/kg) of E161111 (ED_50_ = 2.3) was lower than that of etomidate (ED_50_ = 0.75). The infusion rate (in mg/kg·min) for E161111 was 4.74 ± 0.29, and was 0.25 ± 0.01 for etomidate, and 0.79 ± 0.06 for propofol (Table 2).

**Table 2:** Pharmacodynamic characteristics of E161111, etomidate, and propofol after administration of one bolus and 1-h infusion in rats.

Drugs	One bolus^a^ (*n* = 10 rats each group)				1 h infusion (*n* = 6 rats each group)			
	ED_50_ (mg/kg)	LORR time (min)	Recovery time (min)	Myoclonus (%)	Infusion rate (mg/kg·min)	LORR time (min)	Recovery time (min)	Myoclonus (%)
E161111	2.3	1.39 ± 0.31^#^^*^	0.19 ± 0.10^#^	0	4.75 ± 0.29	1.8 ± 0.3^#^^*^	4.3 ± 1.5^#^^*^	83
Etomidate	0.75	6.83 ± 2.77^*^	1.8 ± 1.63	20	0.25 ± 0.01	27.4 ± 4.1^*^	41.2 ± 6.8^*^	67
Propofol	5.9	12.47 ± 2.64^#^	0.73 ± 0.58	0	0.79 ± 0.06	12.5 ± 2.97^#^	14.5 ± 2.88^#^	0

**Notes:**

a One bolus administration at 2*ED_50_.

* *P* < 0.05 *vs* propofol.

# *P* < 0.05 *vs* etomidate.

ED_50_, median effective dose.

LORR, loss of righting reflex.

Recovery time, recovery from righting reflex to stand up.

### Recovery time after one bolus administration and 1-h infusion

After one bolus administration of the test drug at a dose of 2*ED_50_, the duration (in min) of LORR for E161111 (1.39 ± 0.31) was shorter than that in the two groups: 12.47 ± 2.63 for the propofol group and 6.83 ± 2.77 for the etomidate group. The recovery time (*i.e*., the time needed to be able to stand) after one bolus administration of propofol, E161111 or etomidate, all at 2*ED_50_, was 0.73 ± 0.58, 0.19 ± 0.1 and 1.8 ± 1.63 min, respectively. No myoclonus was observed on rats after one bolus administration of E161111, while myoclonus incidence rate was 20% for etomidate. After 1-h infusion of propofol, E161111 or etomidate, the recovery time (in min) was 14.5 ± 2.88, 4.3 ± 1.5 and 41.2 ± 6.8 (*P* < 0.05), respectively. Hence, the recovery time after 1-h infusion in the E161111 group was shorter than that in the other two groups (*P* < 0.05). We observed the myoclonus incidence rate was 83% for E161111, and 67% for etomidate (Table 2).

### MAP change during 0.5-h infusion and 10 min after infusion

Rats were placed in a telemetry box for 30 min to obtain the MAP at baseline, as well as 1, 5, 10, 15, 25 and 30 min during 0.5-h infusion and 5, 10 min after infusion. Propofol, etomidate, or E161111 were given at their 1.2*ED_50_ for LORR followed by a 0.5-h infusion. The infusion rate for E161111 was 4.78 ± 0.41 mg/kg, 0.82 ± 0.04 mg/kg for propofol and 0.26 ± 0.01 mg/kg for etomidate. In the E161111 group, the MAP was maintained at a steady level: almost no decrease was observed.

**Figure 4:**
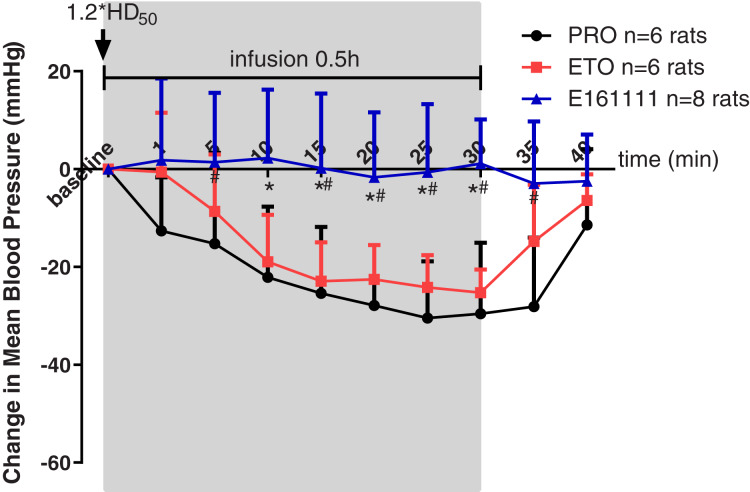
Mean arterial pressure (MAP) during 30-min infusion and 10-min after infusion of propofol (*n* = 6 rats), etomidate (*n* = 6 rats), or E161111 (*n* = 8 rats). Each data represented the average (±standard deviation) MAP change after bolus administration at 1.2*ED_50_ for LORR followed by 0.5-h infusion in rats. The infusion rate for E161111 was 4.78 ± 0.41 mg/kg, 0.82 ± 0.04 mg/kg for propofol (PRO) and 0.26 ± 0.01 mg/kg for etomidate (ETO). (*P < 0.05 *vs* etomidate group, ^#^*P* < 0.05 *vs* propofol group).

However, we documented a MAP decrease for ≥20 min during infusion of propofol or etomidate at equal hypnotic doses. A decrease in MAP was observed in the propofol group (the maximum decreased MAP was −30.46 ± 11.61 mm Hg) and etomidate group (the maximum decreased MAP was −25.25 ± 4.73 mm Hg) from 10-min infusion to 30-min infusion (Fig. 4). The changes in MAP decreased by propofol were significantly lower than those decreased by E161111 from 5 min during 0.5-h infusion to 5 min after infusion (*P* < 0.05). The result is in accordance with data for single injection with etomidate or propofol described in our previous article (Wang et al., 2017). After 0.5-h infusion, the MAP recovered in 10 min.

### Plasma corticosterone concentration after 1-h infusion of E161111: comparisons with etomidate and saline

**Figure 5:**
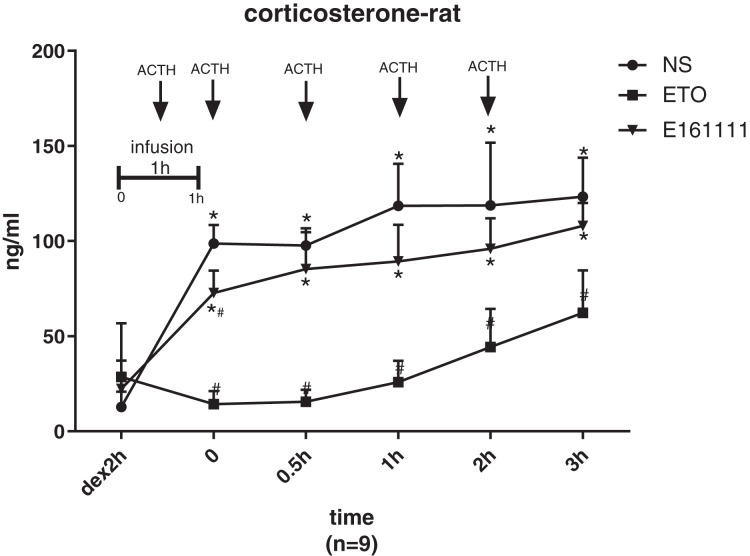
The serum concentration of corticosterone. The serum corticosterone concentration of rats after 1-h infusion of etomidate, E161111 and or saline (*N* = 9 rats for each group). No significant differences were observed between at baseline of three groups. (*P < 0.05 *vs* etomidate (ETO) group, ^#^*P* < 0.05 *vs* Normal Saline (NS) group).

There were no significant differences in the corticosterone concentration at baseline of rats in the etomidate, E161111, or saline groups after dexamethasone inhibition. The corticosterone concentration was lower after infusion of E161111 or saline at all time points for 3 h after infusion. After 1-h infusion, the serum corticosterone concentration (in ng/ml) in the E161111 group (72.69 ± 11.83) was much higher than that in the etomidate group (14.28 ± 6.88, *P* < 0.05), and this effect lasted for 3 h. The serum corticosterone concentration (in ng/ml) after 1-h infusion with E161111 (85.31 ± 19.37) was slightly lower than that in the saline group (134.22 ± 43.07, *P* < 0.05), but it recovered rapidly in 30 min (Fig. 5).

## Discussion

In the present study, we observed the advantages of E161111. It was metabolised rapidly, enabled quick recovery from sedation, showed hemodynamic stability, and revealed little suppression of adrenocortical function after 0.5-h or 1-h continuous infusion. As expected, the main metabolite of E161111 was etomidate acid (which is the main metabolite of etomidate).

The imidazole carboxylic acid ester side chain of etomidate has been reported to influence the GABA receptor (which is the major action site of etomidate) and adrenal steroidogenesis (Atucha et al., 2009). Our previous investigations on ET-26 hydrochloride demonstrated that modifying the ester side chain is a feasible strategy to find better etomidate analogues. To avoid the toxicity of metabolites, the main metabolites of these compounds were designed to be the same as those of etomidate. Etomidate has been used clinically for more than 40 years, and used in ICU for a long time. Hence, its metabolite, etomidate acid, should be safe. Etomidate acid is the primary metabolic product of etomidate, which has less inhibition of the steroid 11β-hydroxylase, another important site that affects the adrenal steroidogenesis (Zolle et al., 2008). To achieve the purpose of quick metabolism and be suitable for infusion, we designed soft drugs from aspect of pharmacokinetic strategy.

E161111 was one of the compounds designed on the basis of this concept. *In vitro* experiments showed that the E161111 was metabolized rapidly in rat plasma and liver homogenate, and the main decomposition product was etomidate acid. After one bolus of administration, E161111 seemed to offer light sedation, unlike the depth of sedation noted after administration of etomidate, propofol. In addition, rats recovered 0.19 ± 0.10 min after administration of E161111 as a single bolus at its 2*ED_50_, almost 1/20 after given etomidate and 1/4 after given propofol. E161111 might be a promising compound for use in the ICU.

A hypnotic compound used in the ICU should have a short duration of action, be metabolised rapidly, enable quick emergence after anaesthesia, and cause no or little haemodynamic/cortical depression. To observe if E161111 was suitable for ICU sedation, we continued our study on the effects of test compounds after continuous infusion.

For patients receiving mechanical ventilation in the ICU, an appropriate target is a RASS score of −2 to 0 (Reade & Finfer, 2014). We adjusted the infusion rate so that rats could be awaken with eye opening to voice, which denotes light sedation. We adjusted the infusion rate to maintain a light sedation level. The highest infusion rate was recorded in the E161111 group. That is, a greater dose of drug must be given to maintain the same depth of sedation due to its low potency. The rats which we infused with E161111 recovered more quickly than rats given the other two test drugs (propofol or etomidate).

Telemetry was used to observe MAP changes in rats. Telemetry technology can exclude the influence of anaesthetics on blood pressure, which is consistent with clinical practice. A decrease in blood pressure was not observed during E161111 infusion. This result indicates that E161111 may have advantage in maintaining cardiovascular stability especially after infusion.

One the most important characteristics we wished to improve was corticosterone suppression. We used exogenous ACTH to stimulate steroidogenesis. After one hour infusion, the stimulated serum corticosterone concentration in the E161111 group was much higher than that in the etomidate group and the lack of inhibitory effect persisted for the three hours’ study. The serum corticosterone concentration after 1-h infusion with E161111 was slightly lower than that in the saline group, but it recovered rapidly in 30 min. This study confirmed our hypothesis that modifying ester side chain may be an effective approach to obtaining etomidate analogues with lower corticosteroid suppression.

The main limitation of our study was that we did not examine the pharmacological effects of E161111 during longer time infusion or on higher species. However, as E161111 is less potent than etomidate and metabolized much more quickly, very large doses of E161111 would need to be infused, producing plasma etomidate acid concentrations that would be much higher than would be reached with etomidate infusions. It may potentially induce unknown side effects. We need do further examination on higher doses and species. The main corticosteroid in humans is cortisol, and the effect of the inhibition would need to be evaluated in clinical settings.

## Conclusions

E161111 was metabolised rapidly, having the same major metabolite as etomidate, and the recovery time after 1-h infusion was short. It elicited haemodynamic stability and milder suppression of corticosterone than that elicited by etomidate.

## Supplemental Information

10.7717/peerj.13492/supp-1 Supplemental Information 1 The ARRIVE guidelines 2.0: author checklist.

10.7717/peerj.13492/supp-2 Supplemental Information 2 Raw data.

## Additional Information and Declarations

### Data Availability

The following information was supplied regarding data availability: The raw data is available in the Supplemental File.
